# Monte Carlo investigation of PET [^68^Ga]Ga-DOTA-TOC activity-administration protocols for consistent image quality

**DOI:** 10.1016/j.heliyon.2023.e19504

**Published:** 2023-08-25

**Authors:** Philip Kalaitzidis, Johan Gustafsson, Cecilia Hindorf, Michael Ljungberg

**Affiliations:** a Medical Radiation Physics, Lund, Lund University, Lund, Sweden; b Department of Medical Radiation Physics and Nuclear Medicine, Karolinska University Hospital, Solna, Stockholm, Sweden

**Keywords:** ^68^Ga]Ga-DOTA-TOC, Anthropomorphic computer phantoms, Monte Carlo simulation, Positron emission tomography

## Abstract

One example of a PET exam that suffers from noise problems is [^68^Ga]Ga-DOTA-TOC, where patients are generally administered between 100 and 200 MBq [^68^Ga]Ga-DOTA-TOC, irrespective of size. However, a fixed activity can result in low signal-to-noise ratios (SNRs) in larger patients. This study aimed to evaluate the impact on image quality with respect to injected activity and patient habitus through Monte Carlo (MC) simulation. Eight anthropomorphic computer phantoms with body mass indices (BMIs) between 19 kg/m^2^ and 38 kg/m^2^ and tumours distributed in the liver were simulated using the MC software Gate v8.2 with an activity distribution defined according to [^68^Ga]Ga-DOTA-TOC standardised uptake values. Three activity-administration protocols were simulated: (i) with a fixed activity of 100 MBq, (ii) with the activity scaled by 2 MBq/kg, and (iii) with the activity scaled by a body size-dependent power-function based on the SNR obtained with (ii). BMI, weight, body surface area, and abdominal circumference were evaluated body size parameters. Images were reconstructed with the CASToR software and evaluated for background SNR and lesion contrast-to-noise ratio (CNR). Large SNR variabilities were obtained with protocols (i) and (ii), while (iii) generated good consistency. Several tumours failed to reach a CNR of 5 for large phantoms with protocol (i), but the CNR was generally improved by (ii) and (iii). An activity scaled by patient habitus generate better image quality consistency, which increases the likelihood that patients receive a similar standard of care.

## 1 Introduction

Monte Carlo (MC) simulations of positron emission tomography (PET) systems can be used to investigate clinically relevant issues that are difficult to evaluate *in vivo* or with physical phantom measurements. The benefit of MC simulations is its ability to isolate the problem and exclude confounders, which has been demonstrated in several previous studies [[1], [2], [3]].

One example of a clinically relevant PET problem that can benefit from MC studies is the problem of image noise, which in turn affects, e.g., detectability. Noise in tomographic images is determined by a combination of the number of detected photons and how noise in projection space propagates to noise in the reconstructed image, both which are affected by the patients’ habitus. Hence, a given injected activity for two different patients may result in completely different noise properties of reconstructed images. As such, the study of noise properties in patient images is hampered by the difficulty of isolating single parameters, while physical phantom measurements offer limited geometrical realism. Monte Carlo simulations with anthropomorphic phantoms may here be a good compromise between the need for a patient-like geometry and the need for controlled experiments.

Neuroendocrine tumours (NETs) constitute a heterogeneous group of malignancies that commonly originate from the gastrointestinal tract [4] with metastases most commonly located in the liver [5]. Most NETs overexpress somatostatin receptors (SSTRs) which enables molecular imaging using somatostatin analogues, e.g., [^68^Ga]Ga-DOTA-TATE and [^68^Ga]Ga-DOTA-TOC [6]. As for all diagnostic imaging, the accuracy of the diagnostic information from ^68^Ga-SSTR imaging depends on the quality of the reconstructed image. However, the PET image quality is affected by several parameters, such as injected activity, patient habitus, acquisition time, and reconstruction settings [7], and may thus vary between patients, in turn leading to an unequal standard of care.

The European Association of Nuclear Medicine (EANM) has published guidelines for PET/CT imaging with ^68^Ga labelled DOTA-conjugates [8] to help in attaining sufficient image quality. The guidelines state: *“The activity administered ranges from 100 to 200 MBq, also depending on the PET scanner technical characteristics and patient body weight. The recommended activity to obtain a good image quality is at least 100 MBq.”* Even if the camera properties and patient weight are mentioned as potential modifying factors for the injected activity, it does not explicitly state to what degree, leaving room for interpretation. An injected activity between 100 MBq and 200 MBq may be sufficient to generate images of good quality for underweight and normal-weight patients, the same may not be true for overweight and obese patients. As patients’ body size increases, the image quality can be expected to decrease as a consequence of increased attenuation and scatter [9]. Furthermore, a lower activity concentration in specific organs can be expected for a fixed injected activity as organ volumes tend to be larger in larger patients, further worsening the image signal-to-noise ratio (SNR).

Accordingly, more effort is needed to evaluate how injected activity and patient habitus affect image quality in [^68^Ga]Ga-DOTA-TOC PET exams. Further understanding of the relationship between injected activity and patient habitus may facilitate obtaining consistent image quality for tumour detection, irrespective of the patient's body size. The problem of achieving consistent image quality in PET has been addressed in some previous studies [[10], [11], [12]]. In a study by de Groot et al. [10] inter-patient image quality consistency for [^18^F]FDG was evaluated and a quadratic relation between administered activity and patient weight was suggested to improve image quality consistency. In similarity with the study by de Groot et al. [10], Cox et al. [12] evaluated inter-patient image quality consistency for [^68^Ga]Ga-DOTA-TATE and showed that better inter-patient image quality consistency was achieved by non-linear scaling of the time-activity product as a function of patient weight. However, evaluating such information can be difficult *in vivo* as the effects of, e.g., motion, and variation in inter-patient radiopharmaceutical uptake, all contribute to a degradation in image quality and cannot be separated from the studied phenomenon, i.e., image noise, in a clinical PET acquisition.

In this study, MC simulated PET images of anthropomorphic computer phantoms are used to study noise and injected activity in ^68^Ga SSTR imaging with [^68^Ga]Ga-DOTA-TOC. Monte Carlo simulations combined with anthropomorphic computer phantoms can be used to systematically examine how patient habitus and scanner settings impact the resulting PET image. We believe such information can facilitate the image interpretation process with clearer guidance on how to improve image quality and receive better inter-patient image quality consistency.

## 2 Materials and methods

The pipeline connecting simulated data to reconstructed images used in this study has been described in a previous study [13]. The PET model consists of 34 detection modules arranged in a ring with a face-to-face diameter of 744.2 mm. Every detection module consists of four axial units, each containing four transaxial detection blocks. Each detection block consists of an array of 4 × 9 transaxial by axial lutetium-yttrium oxyorthosilicate (LYSO) scintillating crystals, and each crystal has the dimensions 5.3 × 3.95 × 25 mm^3^. Energy discriminators were set to 425 keV (lower) and 650 keV (upper), and the total coincidence timing window (2τ) was set to 4.9 ns. A Gaussian energy blurring with a full width at half maximum (FWHM) of 9.63% at 511 keV and a Gaussian temporal resolution with a FWHM of 268.7 ps was applied to each detected singles event. A detection efficiency of 98% was applied to account for crystal transfer efficiency and quantum efficiency. Background signal originating from the radioactive decay of ^177^Lu within the LYSO crystals was modelled with a frequency of 1142 kHz. More details can be found in Kalaitzidis et al. [13].

This study was divided into two parts: validation of the PET model for ^68^Ga imaging by comparison with measurement of the NEMA IQ phantom, and simulations utilising anthropomorphic computer phantoms of varying sizes, generated from the XCAT-population [14,15]. The anthropomorphic computer phantoms and realistic activity distributions enable the replication of clinical PET [^68^Ga]Ga-DOTA-TOC exams.

### 2.1 Validation of the PET model for ^68^Ga

A measurement was performed with the NEMA IQ phantom (volume 9.7 L) on GE's four-ring Discovery MI PET/CT scanner, with a 20 cm axial field-of-view. The system sensitivity of the clinical camera has previously been measured to 13.9 cps/kBq centrally and 12.7 cps/kBq at the 10 cm radial offset. A set of six spheres with diameters of 37 mm, 28 mm, 22 mm, 17 mm, 13 mm, and 10 mm was used. The background compartment and spheres were filled with a ^68^Ga water-solution. The activity concentration in the background compartment was 10.3 kBq/mL, and the sphere activity concentration was 41.0 kBq/mL, i.e., a sphere-to-background ratio of approximately 4:1. The NEMA IQ phantom was positioned with its spheres located on the axial sensitivity peak. Acquisition of data was performed for 3 min for a single bed position. A simulation of the measurement was replicated in the MC program Gate v8.2 [16]. The ^68^Ga source was modelled with the radioactive decay as the starting point (Gate ion source type), physics models for photon and charged particle interaction were set to the emstandard_opt3 physics list, and production cuts were set to 1 mm for all regions.

### 2.2 Simulations of [^68^Ga]Ga-DOTA-TOC with anthropomorphic phantoms

**Fig. 1:**
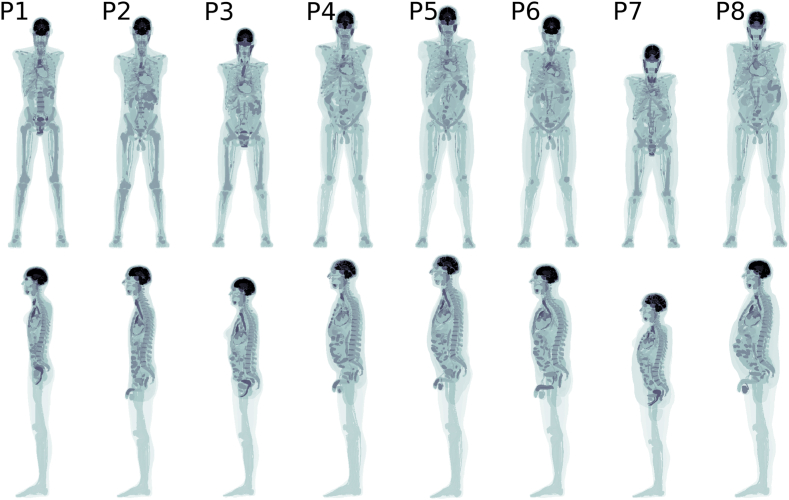
Coronal and sagittal MIPs of the anthropomorphic computer phantoms, where each phantom structure has been assigned a unique code. The phantoms are ordered from left to right with ascending BMI. The computer phantoms' BMI were 19 kg/m^2^, 23 kg/m^2^, 25 kg/m^2^, 28 kg/m^2^, 30 kg/m^2^, 33 kg/m^2^, 37 kg/m^2^, and 38 kg/m^2^.

Eight anthropomorphic computer phantoms from the XCAT-population [14,15] were generated with body mass indices (BMIs) ranging between 19 kg/m^2^ and 38 kg/m^2^. Each computer phantom was generated as a 256 × 256 × 735 image matrix with a voxel size of 2.73 × 2.73 × 2.79 mm^3^. The phantoms were generated without arms to resemble an acquisition with arms placed above the head. Coronal and sagittal maximum intensity projections (MIPs) of the computer phantoms are shown in Fig. 1.

The generated computer phantoms' characteristics, including sex, height, weight, BMI, abdominal circumference, and body surface area (BSA) are presented in Table 1. Abdominal circumference was determined as the largest circumference of each phantom's abdomen and BSA was estimated according to the Du Bois formula [17].

**Table 1:** Physical characteristics of simulated anthropomorphic phantoms.

	Sex	Height [m]	Weight [kg]	BMI [kg/m^2^]	Circumference [cm]	BSA [m^2^]
P1	Female	1.73	56	19	72	1.67
P2	Male	1.74	68	23	84	1.81
P3	Female	1.65	69	25	97	1.76
P4	Male	1.79	89	28	115	2.08
P5	Male	1.82	99	30	113	2.20
P6	Male	1.75	100	33	114	2.15
P7	Female	1.53	86	37	117	1.83
P8	Male	1.79	120	38	138	2.36

The anthropomorphic computer phantoms were generated as code images, with each code uniquely representing an anatomical structure. Each structure was assigned a material during the simulations through a look-up table.

Similarly, a second look-up table was created to assign the activity. The activity concentration and distribution were defined based on clinical and published standardised uptake values (SUVs) [[18], [19], [20], [21], [22], [23], [24], [25]]. In particular, the bladder SUV was based on clinical SUVs of patients emptying their bladder 15 min prior to imaging. The activity in each voxel ($\left[Bq\cdot {vox}^{-1}\right]$) was determined according to

(1) $$\begin{array}{c} A_{vox}=\frac{A_{inj}\cdot SUV_{vox}\cdot V_{vox}}{w_{T}}, \end{array}$$

where *A*_inj_ is the injected activity, *SUV*_vox_ is the SUV recorded approximately 60 min between administration and imaging of the structure associated with the voxel, *V*_vox_ is the voxel volume, and *w*_T_ is the phantom weight. Table 2 lists assigned density and *SUV*_vox_ of phantom structures with a *SUV*_vox_ higher than 5.

**Table 2:** Assigned density and SUV for a few selected phantom structures.

Structure	Density [g/cm^3^]	*SUV*_vox_
Bladder contents	1.00	12.0
Kidneys	1.05	9.7
Liver	1.06	9.5
Pancreas	1.04	5.5
Spleen	1.06	23.2
Stomach wall	1.05	7.0
Tumours	1.05	22.9

Each phantom was simulated thrice with the injected activity, *A*_inj_, altered between each simulation to represent various activity-administration protocols. The three activity-administration protocols simulated were: protocol (i) where *A*_inj_ was set to a fixed activity of 100 MBq, protocol (ii) where *A*_inj_ was scaled with phantom weight by 2 MBq/kg according to the clinical protocol for [^68^Ga]Ga-DOTA-TOC PET imaging at Skåne University Hospital, Lund, and protocol (iii) where *A*_inj_ was scaled similarly to the method proposed by de Groot et al. [10], based on the liver SNR obtained with protocol (ii).

Since the signal in projection space in PET follows Poisson count statistics, the standard deviation is equal to the square root of the signal, i.e., proportional to the square root of the time-activity product. It is then reasonable to assume that, to a first approximation, the standard deviation in the reconstructed image also scales with the square root of the time-activity product [10,12]. Therefore, the liver SNR obtained with protocol (ii) was normalised by $\sqrt{A_{inj}\cdot t}$ to yield a normalised SNR independent of scan acquisition time and injected activity, according to

(2) $$\begin{array}{c} SNR_{Norm}=\frac{SNR_{L}}{\sqrt{A_{inj}\cdot t}}, \end{array}$$

where *SNR*_L_ is the liver SNR obtained with protocol (ii), and *t* is the scan acquisition time per bed position. The normalised SNR was plotted as a function of several body size parameters to find an appropriate body size metric to scale the activity with. The normalised SNR, with respect to a body size parameter, was fitted with a power function according to

(3) $$\begin{array}{c} SNR_{Fit}=a\cdot p^{-d}, \end{array}$$

where *a* and *d* are coefficients determined from a least-squares fit of *SNR*_Norm_ with respect to the evaluated phantoms' body size parameters, *p*. The body size parameters studied were BMI, weight, abdominal circumference, and BSA. The combination of Eqs. (2), (3) forms an expression by which a constant liver SNR can be obtained as,

(4) $$\begin{array}{c} SNR_{Const}=a\cdot p^{-d}\cdot \sqrt{A_{inj}\cdot t}, \end{array}$$

the subsequent rearrangement of Eq. (4) defines the activity-administration protocol needed to achieve, in theory, a constant *SNR*_L_, irrespective of patient habitus, as

(5) $$\begin{array}{c} A_{inj}=\frac{1}{t}\cdot \left(\frac{SNR_{Const}^{2}}{a^{2}}\right)\cdot p^{2d}.\; \end{array}$$

The goodness of the fit with respect to studied body size parameter, Eq. (3), was evaluated with the coefficient of determination (*R*^2^), and the body size parameter generating the best fit with *SNR*_Norm_ was chosen to scale the protocol (iii) activity.

A 60-min time window was assumed between injection and start of imaging with physical decay of ^68^Ga during this time period accounted for in the simulations.

**Fig. 2:**
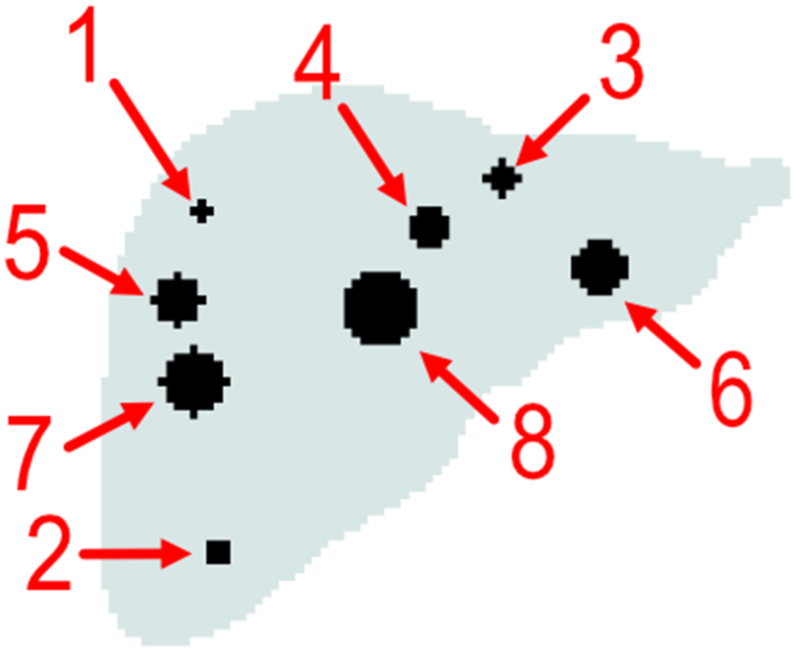
Tumour locations within P8's liver.

Eight tumours were added to the liver of each phantom. The tumours were placed in the liver as 1) NETs commonly metastasise in the liver [26] and 2) because normal liver tissue has a high background concentration of ^68^Ga at the time of imaging. Hence, it is of interest to evaluate tumour-to-liver contrast as detectability may be limited for this organ. Initially, tumour *seeds* were randomly distributed within the liver. A tumour *seed* represents an initial voxel and is the tumour's centre point, each tumour *seed* was then expanded spherically to generate eight tumours of different volumes. The eight tumour volumes were 0.15 mL (tumour 1), 0.40 mL (tumour 2), 0.69 mL (tumour 3), 1.68 mL (tumour 4), 2.56 mL (tumour 5), 3.72 mL (tumour 6), 5.34 mL (tumour 7), and 8.09 mL (tumour 8). Fig. 2 shows the location of tumours within P8's liver.

Uniform activity uptake was defined for the tumours, and the activity level was determined by Eq. (1) using the SUV reported for [^68^Ga]Ga-DOTA-TOC NET liver metastases [27]. Similar tumour placement between phantoms was maintained by reusing the initial tumour *seeds*’ relative position within the liver from one phantom to another.

Simulations were performed over several bed positions starting from mid-thigh to the forehead, and a 46 mm axial overlap was introduced between each bed position. Data were acquired for 3 min per bed position. Computational times were decreased by simulating only a subsample of the whole computer phantom at each bed position, in which particles expected to contribute little or not at all to the acquisition are removed from the simulation. The phantom subsample extended 5 cm beyond each side of the scanner's axial field-of-view. The same source type, physics list, and production cuts were used during the simulations of the anthropomorphic phantoms as for the NEMA phantom simulation. The activity was adjusted between each bed position by physical ^68^Ga deacy, but without biological redistribution or excretion. Phantom organ motion was not included in the simulations.

### 2.3 Image reconstruction

Measured NEMA IQ data were reconstructed with the PET Toolbox™ (GE Healthcare) using ordered-subset expectation-maximisation (OS-EM) with time-of-flight (TOF) information included, using 3 iterations and 16 subsets and post-filtered with a 5.5 mm FWHM Gaussian kernel transaxially and GE's standard z-filter axially. The simulated NEMA IQ data were reconstructed with the CASToR software [28] using OS-EM with TOF information included, using 3 iterations and 16 subsets and post-filtered with a 5.5 mm transaxial and 4 mm axial FWHM Gaussian kernel. Both reconstructions included compensation for attenuation, normalisation, randoms, and scatter. The measured data also included deadtime and pile-up corrections.

The anthropomorphic computer phantoms were reconstructed with CASToR using the same settings as for the NEMA phantom simulation.

### 2.4 Evaluation of the PET model for ^68^Ga

Transverse slices of the NEMA IQ phantom from the measurement and simulation were visually compared. Additionally, a profile was drawn over the largest (Ø = 37 mm) and third smallest (Ø = 17 mm) NEMA IQ spheres for the measured and simulated reconstructed images.

Signal-to-noise ratios and contrast-to-noise ratios (CNRs) were evaluated for each NEMA sphere according to

(6) $$\begin{array}{c} SNR_{i}=\frac{{\bar{x}}_{B,i}}{\sigma_{B,i}}, \end{array}$$

and

(7) $$\begin{array}{c} CNR_{i}=\frac{{\bar{x}}_{S,i}-{\bar{x}}_{B,i}}{\sigma_{B,i}}, \end{array}$$

where ${\bar{x}}_{B,i}$ is the mean activity concentration of 60 regions-of-interest (ROIs) placed in the background compartment and $\sigma_{B,i}$ is the standard deviation over all voxels within the 60 background ROIs. The index *i* signifies the diameter of the ROIs, matching the diameters of NEMA spheres. The placement of the background ROIs was determined according to the NEMA NU 2–2007 standard [29]. The mean sphere activity concentration, ${\bar{x}}_{S,i}$, was determined from ROIs placed over the evaluated sphere.

### 2.5 Evaluation of [^68^Ga]Ga-DOTA-TOC simulations with anthropomorphic phantoms

Liver SNR and tumour-to-liver CNR were evaluated for each activity-administration protocol and anthropomorphic computer phantom. The liver SNR was measured according to

(8) $$\begin{array}{c} SNR_{L}=\frac{{\bar{x}}_{L}}{\sigma_{L}}, \end{array}$$

where ${\bar{x}}_{L}$ is the mean activity concentration from a spherical (Ø = 25 mm) volume-of-interest (VOI) placed in a uniform part of the liver and σ_L_ is the voxel-to-voxel standard deviation of the activity concentration in the spherical VOI. The CNR was calculated according to

(9) $$\begin{array}{c} CNR=\frac{{\bar{x}}_{T}-{\bar{x}}_{L}}{\sigma_{L}}, \end{array}$$

where ${\bar{x}}_{T}$ is the mean activity concentration defined for a VOI encompassing each tumour, ${\bar{x}}_{L}$ and $\sigma_{L}$ were determined from the same spherical VOI that was used to determine *SNR*_L_ in Eq. (8).

## 3 Results

### 3.1 Validation of the PET model for ^68^Ga

**Fig. 3:**
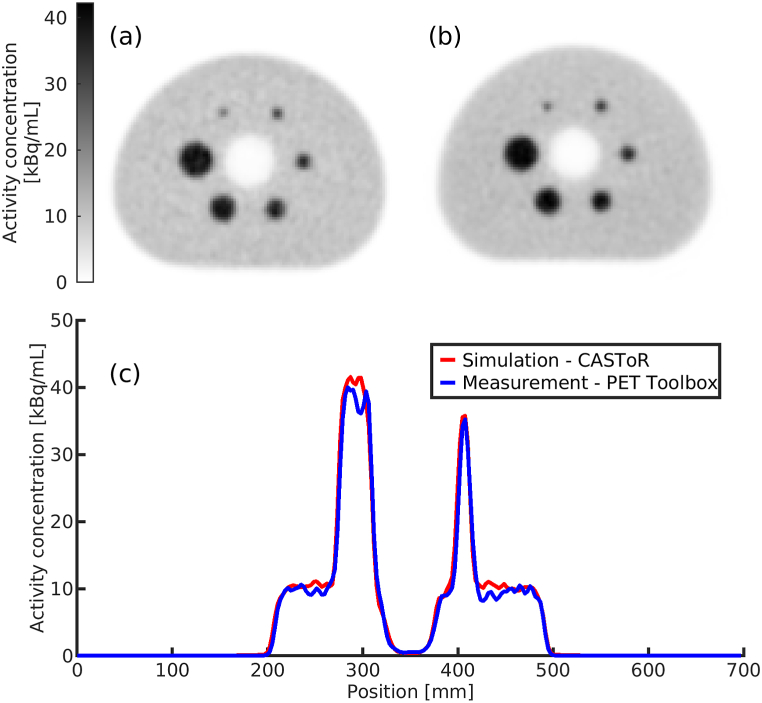
Transverse slice of the measured NEMA IQ phantom reconstructed with the PET Toolbox (a) and corresponding transverse slice from the simulation reconstructed with CASToR (b). Profiles through the largest (Ø = 37 mm) and third smallest sphere (Ø = 17 mm) are shown in (c).

**Fig. 4:**
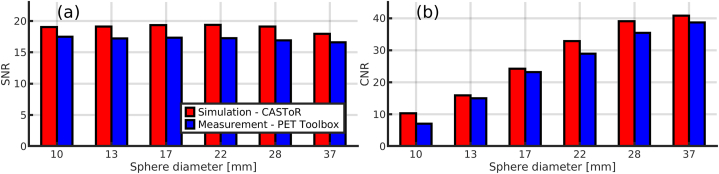
Signal-to-noise ratio (a) and contrast-to-noise ratio (b) of the simulated and measured NEMA IQ phantom.

Transverse reconstructed slices from the measurement and simulation of the NEMA IQ phantom with profiles through the largest (Ø = 37 mm) and thirds smallest (Ø = 17 mm) spheres are shown in Fig. 3. The measured image reconstructed with the PET vendor Toolbox appear slightly noisier (a) than the corresponding simulated image reconstructed with CASToR (b). Profiles through the largest and third smallest sphere match well between the measurement and simulation (c). Signal-to-noise ratios and CNRs (Eqs. (6) and (7)) are shown in Fig. 4, with both the SNR and CNR moderately higher in the simulated phantom compared to the measured.

### 3.2 Simulations of [^68^Ga]Ga-DOTA-TOC with anthropomorphic phantoms

**Fig. 5:**
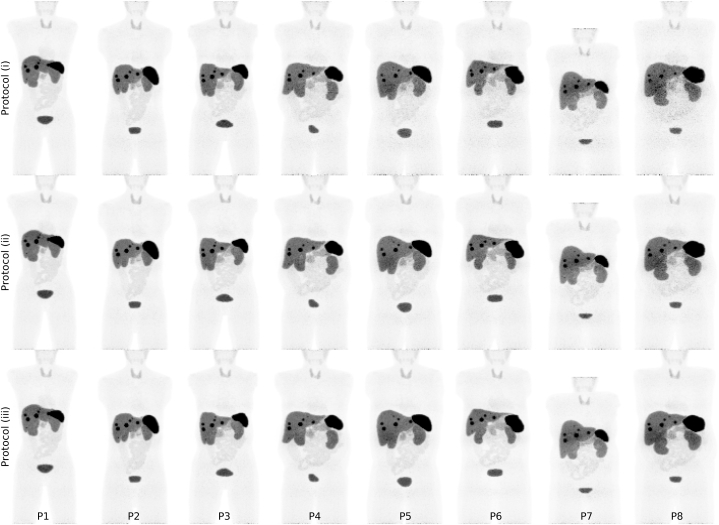
MIPs of PET images, simulated with activity-administration protocols (i), (ii), and (iii). For larger phantoms, the presence of noise is more prominent when protocol (i) is utilised. Protocols (ii) and (iii) reduce the presence of noise, and the images appears to exhibit more similar inter-phantom noise properties, irrespective of patient habitus.

Maximum intensity projections of reconstructed images simulated with protocols (i), (ii), and (iii) are shown in Fig. 5.

**Fig. 6:**
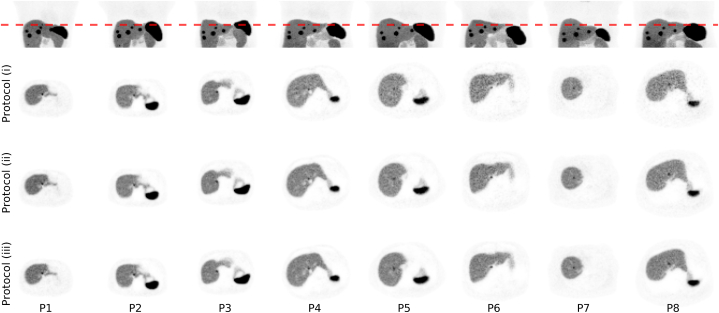
Transverse slices over the liver containing tumour 3 (0.69 mL). The MIPs (upper row) are used to indicate which slice the transverse images display.

Transverse images simulated with protocols (i), (ii), and (iii) are shown in Fig. 6. For larger phantoms, there are large variations in perceived noise properties between protocols. Visually, the larger phantoms, e.g., P8, exhibit larger presence of noise with protocol (i), compared to both protocols (ii) and (iii). For the thinner phantoms, e.g., P1, P2, and P3, the protocol does not appear to substantially alter perceived image quality.

**Fig. 7:**
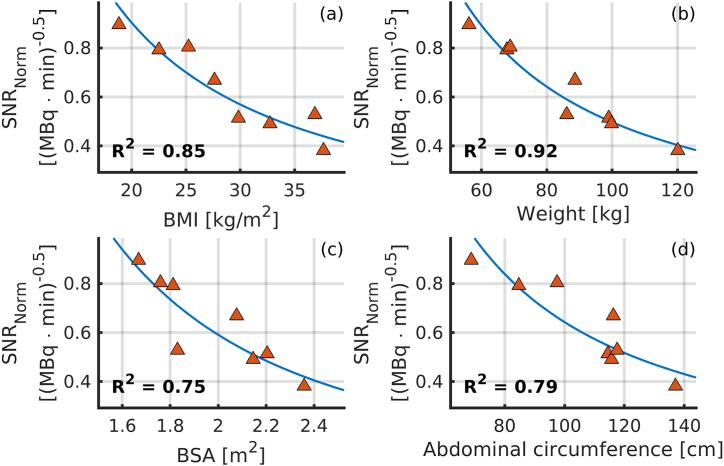
*SNR*_Norm_ and SNR_Fit_ obtained with Eqs. (2) and (3) for simulations performed with protocol (ii), shown as function of BMI (a), weight (b), BSA (c), and abdominal circumference (d).

Normalised SNR as a function of BMI, weight, BSA, and abdominal circumference is shown in Fig. 7. *R*^2^ was determined to 0.85 for BMI (a), 0.92 for weight (b), 0.75 for BSA (c), and 0.79 for abdominal circumference (d). The fit of the normalised SNR as a function of weight generated the highest *R*^2^ value. Fit parameters *a* and *d* with respect to weight were determined to 97.40 and 1.15, respectively. Table 3 lists the activity administered with each protocol.

**Table 3:** Activity administered with the three protocols 60 min prior to imaging. Activity administered with protocol (iii) is determined for a 3-min acquisition per bed position, with weight as body size parameter, and with a constant *SNR*_L_ of 15.

	Protocol (i) [MBq]	Protocol (ii) [MBq]	Protocol (iii) [MBq]
P1	100	112	81
P2	100	136	125
P3	100	138	129
P4	100	177	230
P5	100	198	297
P6	100	200	302
P7	100	172	215
P8	100	240	462

**Fig. 8:**
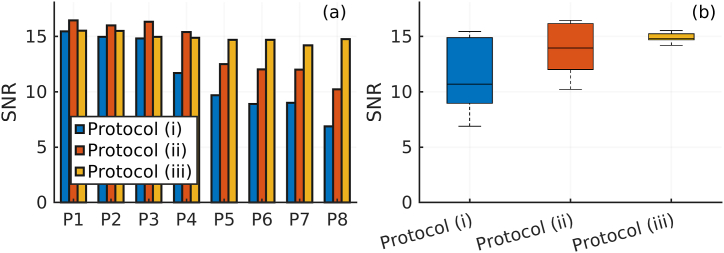
SNR in the liver of the anthropomorphic computer phantoms. The VOI in each phantom is placed in a uniform region of the liver. The large *SNR*_L_ differences are reduced with protocol (iii), compared to protocols (i) and (ii), as seen in both the bar graph (a) and in the boxplots (b).

Bar plots of *SNR*_L_ (Eq. (8)) for protocols (i), (ii), and (iii), are shown in Fig. 8 (a). The *SNR*_L_ for protocols (i) and (ii) decreases substantially as phantom size increases, and the decrease becomes most prominent after phantom 3 and higher for protocol (i). A median *SNR*_L_ of 10.7 and a range (maximum – minimum) of 8.6 was obtained with protocol (i). A gradual *SNR*_L_ decrease is observed with protocol (ii), but the decrease is not as large as with protocol (i). Protocol (ii) yielded a higher *SNR*_L_ for all phantoms but did not improve inter-patient *SNR*_L_ consistency considerably. Protocol (ii) exhibits a median *SNR*_L_ of 14.0 and a range of 6.2. The most consistent *SNR*_L_ was obtained with protocol (iii), with a range of 1.3 and a median of 14.8. Fig. 8 (b) shows boxplots of *SNR*_L_, demonstrating the improved inter-patient *SNR*_L_ consistency of protocol (iii) compared to both protocols (i) and (ii).

**Fig. 9:**
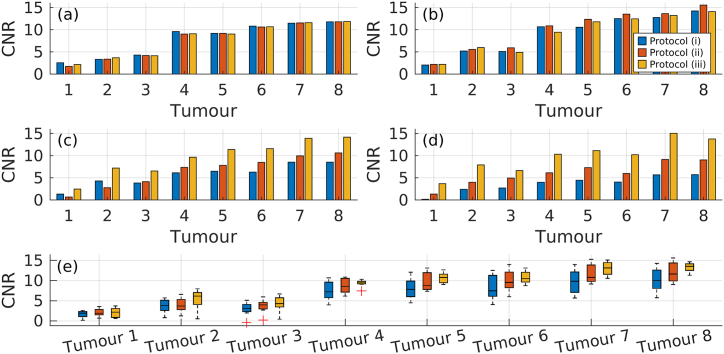
Tumour-to-liver CNR is shown for phantoms P1 (a), P3 (b), P5 (c), and P8 (d). Boxplots of all tumours are shown in (e), with red crosses indicating outliers.

**Fig. 10:**
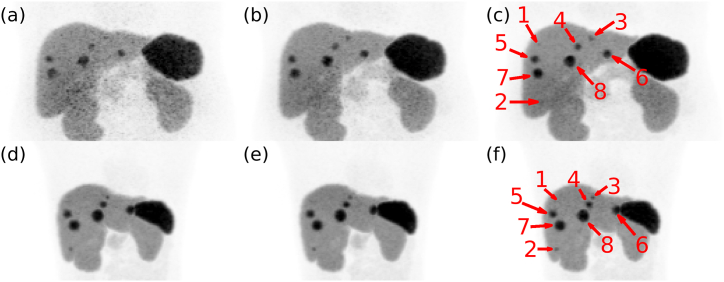
The eight tumours distributed in the liver are shown pointed at by the red arrows. Sub-figures (a), (b), and (c) show reconstructed images for phantom P8 simulated with protocols (i), (ii), and (iii), respectively. Sub-figures (d), (e), and (f) show reconstructed images for phantom P1 simulated with protocols (i), (ii), and (iii), respectively.

The CNR (Eq. (9)) exhibits a substantial decrease with protocol (i) as phantom body size increases, and for the larger phantoms the CNR falls below 5 for several of the tumours. For the same tumours, the CNR is increased substantially with protocols (ii) and (iii). The issue is more prominent the smaller the tumour and the larger the phantom is, but with protocol (iii), the CNRs of the larger phantoms' tumours are elevated to a comparable level to the CNRs of the smaller phantoms’ tumours, as conveyed in Fig. 9 (a–d). The low CNR for certain tumours is reflected in the difficulty to detect those tumours, as seen in Fig. 5, Fig. 6, Fig. 10. In some cases, the CNR falls below 5, further decreasing the reliability of interpretation, i.e., falling below the Rose criterion [30]. The trend is a higher median CNR and generally a smaller range for both protocols (ii) and (iii) compared to protocol (i), as conveyed in Fig. 9 (e). The higher CNR and smaller range increase the reliability that a signal seen after reconstruction originates from a true lesion rather than noise.

Maximum intensity projections covering P1 and P8's livers are shown in Fig. 10. The low CNR obtained for P8 with protocol (i) makes reliably distinguishing tumours from the background more difficult. P8's tumours are visually easier to distinguish with protocol (ii) and even easier with protocol (iii) compared to protocol (i). Similar perceived image quality is achieved with protocol (iii) for phantoms P1 and P8, as seen in Fig. 10 (c) and (f). The smallest tumour cannot be distinguished from the background, irrespective of administration protocol or phantom, while the second smallest tumour is visible in P1 but not in P8.

## 4 Discussion

While MC simulations can be used to evaluate quantitative and qualitative properties of PET images, the validity of this study is dependent on the quality of the computational chain's PET imaging process. Therefore, the computational chain's ability to simulate clinically realistic ^68^Ga PET acquisitions was assessed by a measurement of the NEMA IQ phantom and a corresponding simulation. The computational chain has previously been thoroughly validated for ^18^F [13], and a less extensive evaluation for ^68^Ga was performed for this study, with good agreement between measurement and simulation (Fig. 3). However, the Gate model does not include deadtime and pile-up modelling; therefore, a higher simulated SNR and CNR is expected compared to a corresponding measurement. Hence, the simulated NEMA IQ image in Fig. 3 (b) appears less noisy than its measured counterpart. On the whole, the results indicate that the computational chain can be utilised for ^68^Ga applications, allowing us to use it for noise properties of [^68^Ga]Ga-DOTA-TOC exams.

The study aimed to evaluate how different activity-administration protocols and patient habitus influence inter-patient image quality. In EANM's published guidelines [8] for SSTR PET imaging, 100–200 MBq [^68^Ga]Ga-DOTA-TOC is recommended to obtain diagnostic images with good quality. However, the activity recommended in the guidelines may be unnecessarily high or insufficiently low for certain patients. For some patients, an activity that exceeds 100 MBq may not necessarily generate more diagnostic information than if less activity was used, and the patient may instead be exposed to unnecessary ionising radiation. Conversely, an inadequate activity could, for other patients, deteriorate the quality of the image, and vital information may be lost. Therefore, the administration of [^68^Ga]Ga-DOTA-TOC should preferably be individually optimised based on each patient's body size. In a study by de Groot et al. [10], a similar attempt to achieve better inter-patient image quality consistency for [^18^F]FDG PET found that non-linearly scaled activity dependent on patient body mass resulted in better SNR consistency compared to an activity scaled linearly by patient weight. Likewise, Cox et al. [12] evaluated image quality consistency for ^68^Ga-DOTA-TATE and also found that body mass was a suitable parameter to scale the time-activity product to obtain a more consistent inter-patient image quality. Correspondingly, we have evaluated image quality consistency for [^68^Ga]Ga-DOTA-TOC by MC simulations of anthropomorphic computer phantoms. Since [^68^Ga]Ga-DOTA-TOC and [^68^Ga]Ga-DOTA-TATE possess similar biodistribution and diagnostic accuracy [27], this study can be complementary to the study by Cox et al. [12] as MC simulations allow for complete control over parameters that are not separable in a clinical measurement, such as tumour burden, tumour size and location, and patient motion.

The simulations of anthropomorphic phantoms aimed to resemble clinical [^68^Ga]Ga-DOTA-TOC PET exams. Therefore, the total activity distributed within the imaged part of the phantoms was compared to the activity recovered from clinical [^68^Ga]Ga-DOTA-TOC images to ensure that the simulated activity was clinically realistic. Activity recovered from eleven clinical [^68^Ga]Ga-DOTA-TOC PET images and the activity within the imaged part of the simulated phantoms were both approximately 40% of the initial injected activity (data not shown).

The diagnostic value of a [^68^Ga]Ga-DOTA-TOC PET exam can be improved if the activity-administration protocol is optimised to patient habitus. Similar to de Groot et al. [10] and Cox et al. [12], weight appeared to be the best body size parameter to scale injected activity for a better image quality consistency. A multi-variate regression could be employed to further adapt the injected activity to the individual patient. However, a multi-variate regression would also complicate the clinical process while not necessarily improving image quality substantially, as mentioned by de Groot et al. [10].

The decreased number of detected coincidences as patient body size increases can be compensated for by considering patient habitus in the activity-administration protocol, in turn, decreasing the standard deviation and increasing the precision of the measurement. Thus, a higher SNR can be assumed to increase the confidence that the observed signal in the reconstructed image reflects the actual radiopharmaceutical distribution rather than noise. The largest SNR variation was obtained with protocol (i). This was expected as the injected activity is the same irrespective of patient habitus. The introduction of protocol (ii) aimed to reduce SNR variability by scaling activity linearly with weight. However, scaling the activity linearly with phantom weight maintained a large SNR variability as the gain in SNR was not substantially higher for the larger phantoms compared to the thinner phantoms. The most consistent SNR was obtained with protocol (iii). The range in SNR obtained with protocols (i), (ii), and (iii) was 8.6, 6.2, and 1.3, respectively. The lower SNR variability obtained with protocol (iii) can be attributed to the large gain in SNR for the larger phantoms. Thus, the higher SNR obtained with protocol (iii) for the larger phantoms can be expected to lead to a decreased risk of false positives.

The CNR reflects the ability to distinguish lesions in the background. Lesion CNR obtained with protocol (i) was considerably lower for the large phantoms compared to the CNR obtained with protocols (ii) and (iii), as indicated in Fig. 9. Furthermore, the Rose criterion states that image features with a CNR less than 5 may be difficult to distinguish from background [30], which highlights the issue of reliably distinguishing tumours with protocol (i) as the CNR for several of the tumours were lower than 5, especially for the largest phantom. With protocols (ii) and (iii), some tumours with a CNR previously below 5 were lifted to a value above the Rose criterion, which, by extension, reduces the risk of false negatives. The ability to elevate the CNR above the rose criterion with protocols (ii) and (iii) in the larger phantoms highlights the importance of adapting the administered activity to each patient.

For phantom P1 in Fig. 10 (d – f), all tumours, except tumour 1, are visible with similar shapes and sizes, and the perceived image quality is similar regardless of which protocol was used. Conversely, some tumours appear different in both shape and size from each other in Fig. 10 (a – c), and each sub-figure appears to have a different amount of perceived noise. Furthermore, tumours 1 and 2 were the smallest tumours with volumes of 0.15 mL and 0.40 mL, respectively, and were not discernible in Fig. 10 (a - c). Tumours 1 and 2 were expected to be difficult to distinguish from the background as small lesions are strongly affected by partial-volume effects [31]. Additionally, tumours 3 and 4 would be harder to discern from noise with certainty in Fig. 10 (a) compared to (c) because of the perceived noise level. Moreover, a varying radiopharmaceutical tissue uptake may further influence the ability to detect tumours. Certain tumours may fail to reach a CNR exceeding the Rose criterion for lower tumour-to-background ratios [32].

A problem with using the simulations for studying noise properties and injected activity is that increased activity, as with protocol (iii), does not necessarily lead to equivalent improvement in SNR and CNR for a real measurement because of deadtime and pile-up. Since these effects are not included in the Gate model, the high activities used in some simulations would likely yield slightly worse SNR and CNR if performed as real studies [33].

The harmonised noise level obtained by protocol (iii) could enhance the diagnostic interpretation by enabling a consistent image confidence level, extendable across patients. Notwithstanding, while SNR consistency is improved with protocol (iii), protocol (ii) produces nearly as many lesions with CNRs above the Rose criterion as protocol (iii). In the absence of any additional benefit in detectability, certain patients can avoid excessive exposure to ionising radiation by opting for protocol (ii). A similar rationale has been adopted by the EANM guidelines for [^18^F]FDG oncological imaging [34], where the time-activity product combined with patient weight is used to determine a minimum administered activity. The [^18^F]FDG guidelines provide recommendations for both a linearly and a quadratically scaled activity, where the quadratic alternative intends to compensate for a lower SNR generally obtained with bigger patients. However, for larger patients, the quadratic scaling alternative results in higher activities and, consequently, higher effective doses. Hence, multiple alternatives for activity prescription could be provided, and the choice of prescription alternative is a balance of maintaining satisfactory image quality and minimising patient radiation exposure.

A limitation of protocol (iii) is that the injected activity exceeds 460 MBq for the heaviest phantom for an acquisition time of 3 min per bed position. For clinical practices, it may be unreasonable to perform [^68^Ga]Ga-DOTA-TOC PET exams with those activities as the activity that can be extracted from the ^68^Ga generator is limited. With an expansion of cyclotron-produced ^68^Ga, the activity output could be much higher [35], and the availability of ^68^Ga activity would be less of an issue. Alternatively, the desired SNR level in Eq. (5) could be decreased from 15 to, for example, 12 and still generate adequate image quality while the injected activity would decrease substantially and, consequently, the effective dose. By tolerating an SNR of, for example, 12, the majority of phantoms herein would be administered activities lower than those generated by protocol (ii). For small phantoms, e.g., P1, the activity would be lower than the threshold suggested by EANM's guidelines, sparing those patients unnecessary exposure to ionising radiation. Notwithstanding, while a general goal in a diagnostic context is to minimise radiation exposure while maintaining adequate image quality, an injected activity higher than the 200 MBq limit suggested by EANM's guidelines may be justifiable to improve image quality consistency, particularly for overweight and obese patients.

A second option would be to increase the acquisition time per bed position. Halpern et al. [9] showed an increased interobserver concordance in lesion detection as the acquisition time per bed position increased for [^18^F]FDG. In the same study by Halpern et al. [9], 64% of all lesions were detected with an acquisition time of 1 min per bed position and complete lesion detection was achieved with an acquisition time of 5 min per bed position. Furthermore, Tateishi et al. [36] evaluated the image quality and quantification accuracy dependence on phantom weight for ^89^Zr and found an improved image quality and quantification accuracy with acquisition time scaled by weight. Hence, increasing the acquisition time per bed position for the large phantoms, e.g., P8, from, e.g., 3 min to 4 min, would lower the activity used with protocol (iii) while maintaining adequate image quality.

Nevertheless, administration of [^68^Ga]Ga-DOTA-TOC based on patient weight improves image quality consistency considerably and reduces the risk that inadequate activity is administered to larger patients. An insufficient activity can result in images of substandard quality where valuable diagnostic information may be lost, a risk that is reduced with protocol (iii). However, protocol (iii) is dependent on the type of PET scanner and reconstruction settings which should be considered before clinical implementation. Furthermore, simulations were performed 60 min post activity administration, but the rationale of constructing protocol (iii) would be the same for other imaging time points. A unified image noise level may facilitate the image interpretation process on decisions of what may be considered as pathology and what may not. Hence, a more consistent inter-patient image quality increases the likelihood that every patient receives the same standard of care, irrespective of their body size.

## 5 Conclusion

Injection of a fixed activity irrespective of the patient's physical habitus tends to deteriorate image quality for larger patients. Efforts to improve image quality consistency between patients can help to improve the diagnostic quality of the image for overweight and obese patients. With a non-linear activity scaling by patient weight in the activity administration-schedule of the radiopharmaceutical, smaller variability in image quality over a range of patient habitus is achievable.

## Ethical statement

The patient data used herein were acquired as part of a study approved by the Regional Ethical Review Board at Lund University (approvals 2016/417 and 2018/753) and was performed in accordance with the Declaration of Helsinki. All patients gave written consent.

## Funding

Michael Ljungberg was supported by Mrs. Berta Kamprad's 10.13039/100015230Cancer Foundation [FBKS 2019–44]; Philip Kalaitzidis was supported by the Royal Physiographic Society of Lund [41843].

## Author contribution statement

Philip Kalaitzidis: Conceived and designed the experiments; Performed the experiments; Analyzed and interpreted the data; Wrote the paper. Johan Gustafsson; Cecilia Hindorf; Michael Ljungberg: Conceived and designed the experiments; Wrote the paper.

## Data availability statement

Data will be made available on request.

## Declaration of competing interest

The authors declare that they have no known competing financial interests or personal relationships that could have appeared to influence the work reported in this paper.
